# Combining Automatic Tube Current Modulation with Adaptive Statistical Iterative Reconstruction for Low-Dose Chest CT Screening

**DOI:** 10.1371/journal.pone.0092414

**Published:** 2014-04-01

**Authors:** Jiang-Hong Chen, Er-Hu Jin, Wen He, Li-Qin Zhao

**Affiliations:** Department of Radiology, Beijing Friendship Hospital, Capital Medical University, XiCheng District, Beijing, China; University of Groningen, Netherlands

## Abstract

**Objective:**

To reduce radiation dose while maintaining image quality in low-dose chest computed tomography (CT) by combining adaptive statistical iterative reconstruction (ASIR) and automatic tube current modulation (ATCM).

**Methods:**

Patients undergoing cancer screening (n = 200) were subjected to 64-slice multidetector chest CT scanning with ASIR and ATCM. Patients were divided into groups 1, 2, 3, and 4 (n = 50 each), with a noise index (NI) of 15, 20, 30, and 40, respectively. Each image set was reconstructed with 4 ASIR levels (0% ASIR, 30% ASIR, 50% ASIR, and 80% ASIR) in each group. Two radiologists assessed subjective image noise, image artifacts, and visibility of the anatomical structures. Objective image noise and signal-to-noise ratio (SNR) were measured, and effective dose (ED) was recorded.

**Results:**

Increased NI was associated with increased subjective and objective image noise results (*P*<0.001), and SNR decreased with increasing NI (*P*<0.001). These values improved with increased ASIR levels (*P*<0.001). Images from all 4 groups were clinically diagnosable. Images with NI = 30 and 50% ASIR had average subjective image noise scores and nearly average anatomical structure visibility scores, with a mean objective image noise of 23.42 HU. The EDs for groups 1, 2, 3 and 4 were 2.79±1.17, 1.69±0.59, 0.74±0.29, and 0.37±0.22 mSv, respectively. Compared to group 1 (NI = 15), the ED reductions were 39.43%, 73.48%, and 86.74% for groups 2, 3, and 4, respectively.

**Conclusions:**

Using NI = 30 with 50% ASIR in the chest CT protocol, we obtained average or above-average image quality but a reduced ED.

## Introduction

Lung cancer is the leading global cause of death due to malignancy, [1], [2] and is usually diagnosed at an advanced stage with a subsequent poor prognosis. However, the 5-year recurrence-free survival rate in patients with stage IA non-small cell lung cancer is as high as 80%, [3] emphasizing the importance of early detection. Low-dose computed tomography (CT) screening was associated with a 20% reduction in lung cancer mortality in the National Lung Cancer Screening Trial (NLST), [4], [5] a large randomized control trial conducted in the United States. However, the radiation burden from CT screenings that need multiple CT exams may be of concern.[6]–[8] For example, in the NLST, three rounds of screening were performed at 1-year intervals, producing an average effective dose (ED) of around 1.5 m Sv every time, [9] with a cumulative radiation dose of 4.5 mSv. Radiologists, therefore, continuously optimize CT scan protocols to reduce dosage while maintaining diagnostic image quality, with manufacturers also improving hardware and software.

Methods to reduce radiation dose including reducing the tube current and peak voltage, as well as increasing gantry rotation and table speed, [10]–[13] but these methods produce higher image noise, leading to poor image quality. Automatic tube current modulation (ATCM) in CT can help achieve good image quality while reducing the overall radiation dose to patients. In one implementation of ATCM, the noise index (NI) is used to control the average image noise level for the study population. Higher NI reduces the radiation dose required but also increases objective image noise. Optimal NI selection makes it possible to achieve clinically acceptable images at the lowest radiation dose to patients. In addition, image noise can be reduced with advanced reconstruction algorithms, such as adaptive statistical iterative reconstruction (ASIR), which is widely used, or the newer model-based iterative reconstruction (MBIR) under the commercial name of Veo, a much more complex and advanced algorithm than ASIR. [14], [15] Unfortunately, Veo is not commercially available in China.

Unlike the standard image reconstruction algorithm, filtered back projection (FBP), ASIR can reduce image noise, maintain spatial resolution and image contrast, and improve image quality. [16], [17] Many previous studies [18]–[23] focused on dose reduction by using iterative reconstruction, in contrast to FBP performed with a single combination of NI and ASIR percentage. Only one anthropomorphic phantom study testing optimal parameters for NI and ASIR percentage for low-dose chest CT screening. [24] In this study, we evaluated the image quality and radiation dose of low-dose chest CT images acquired at various NI settings and reconstructed with different ASIR levels.

## Subjects and Methods

The institutional ethics committee of Beijing friendship hospital-affiliated capital medical university approved this study and a written informed consent was obtained from all subjects.

### Patient Population

From April 15 to November 7, 2012, 200 participants were referred to our department for low-dose chest CT screening. They were separated into 4 groups of different NIs, with 50 patients in each group matched for sex, weight, and height. The study population consisted of 130 male and 70 female subjects (mean age, 52.66 years; range, 29–88 years). No participants had severe respiratory symptoms that interfered with their breath-hold CT scan process. They had ability to lie on their back with arms raised over the head. Patient height and weight were used to calculate body mass index (BMI).

### Scanning Technique

All patients were scanned with a 64-slice, high-definition CT scanner (Discovery HD 750, General Electric Healthcare). The scan range covered the whole lung, from the level of the pulmonary apex to the liver dome. All patients were required to hold their breath during the examination. Image acquisition parameters included collimation, 64×0.625 mm; slice thickness, 5 mm; pitch, 0.984; rotation time, 0.5 s; table speed, 78.75 mm/s; tube voltage, 100 kVp. Tube current modulation in the x, y, and z axes (Auto mA, GE Healthcare) was used, with a tube current range of 10–400 mA. The NI was set at 15 for group 1 as the standard protocol for a chest scan in our department. The NIs were set at 20, 30, and 40 for groups 2, 3, and 4, respectively. After each examination for the patients using NI = 40, images reconstructed with 30% ASIR were immediately reviewed by a radiologist on the operator console for image quality. If non-diagnostic images were obtained, a new study using NI = 30 was subsequently performed. This fact was mentioned in the informed consent.

A set of mixed reconstruction images with different levels of ASIR and FBP were generated, namely, 0% ASIR, 30% ASIR, 50% ASIR and 80% ASIR, in which 0% ASIR means 0% ASIR blended with 100% FBP, 30% ASIR means 30% ASIR blended with 70% FBP, etc. Therefore, each acquisition set had 4 series of images with different ASIR levels.

### Assessment of Image Quality

The four series of reconstructed images from each patient were transferred to a picture archiving and communication system (PACS) workstation (Unisight Version 4.2, DJ HealthUnion Systems Corp) for review. All patient and scanner demographic data were removed. Two radiologists, with 13 and 14 years of experience in assessing thoracic image quality, assessed all image data sets for image quality using mediastinal window (width, 400 HU; level, 40 HU) and lung window (width, 1,500 HU; level, –700 HU). To minimize bias in subjective image quality assessment, single reconstructed series were assessed randomly from different patients.

The lesions (emphysema, solid nodule, ground glass opacity nodule, patchy consolidation, patchy ground glass opacity, scarring and calcification, lesion of pleura, etc.) in the lung of each patient were assessed by two radiologists. Subjective visual lesion conspicuity was assessed on a five-point scale [22] (1, well-seen lesion with well-visualized margins; 2, well-seen lesion with poorly visualized margins; 3, subtle lesion; 4, probably an artifact mimicking a lesion; 5, definite artifact mimicking a lesion). Subjective image quality was assessed in terms of subjective image noise, artifacts, and visibility of anatomical structures. [22] Subjective image noise was evaluated in the mediastinal window and was graded on a 5-point scale (1, minimal image noise; 2, less than average image noise; 3, average image noise; 4, more than average image noise; 5, unacceptable image noise). Image artifacts were assessed in the lung window and were graded on a 4-point scale (1, no artifacts; 2, minor artifacts not interfering with diagnostic decision making; 3, major artifacts affecting visualization of major structures, diagnosis still possible; 4, obvious artifacts affecting diagnostic decision making). The visibility of anatomical structures, including the mediastinum, chest wall, and small structures (peripheral bronchovascular bundles located in the peripheral 2 cm of the lungs) was ranked on a 5-point scale (1, good visibility; 2, above average visibility; 3, average visibility; 4, suboptimal visibility; 5, unacceptable visibility). The mediastinum and chest wall were reviewed in the mediastinal window, whereas the bronchovascular bundles were observed in the lung window.

A 190- to 210-mm^2^ circular region of interest (ROI) was placed in the center of the descending thoracic aorta, without touching the lumen walls, at the level of the sixth thoracic vertebra in the mediastinal window. The mean value (Hounsfield unit [HU]) from the ROI was interpreted as the signal and the standard deviation (SD) as the objective image noise. The signal-to-noise ratio (SNR) was calculated with the mean value divided by SD. Objective image noise and SNR were used to assess objective image quality.

### Estimation of Radiation Dose

The scanner calculates the volumetric CT dose index (CTDI_vol_) and dose-length product (DLP) automatically. We converted the DLP to the ED in millisieverts (mSv) by multiplying it by the thoracic conversion factor of 0.0144 mSv mGy^−1^ cm^−1^. [25].

### Statistical Analysis

Data were analyzed using SPSS for Windows (version 17; Chicago, IL, USA). For quantitative data, the results are expressed as the mean ± standard deviation (SD). One-way analysis of variance (ANOVA) was used to compare the age, sex, height, weight, BMI, CTDI_vol_, DLP, and ED among the 4 groups with different NI values. Objective image noise and SNR were compared between the groups of NI = 30 and NI = 40 using ANOVA with Bonferroni post-hoc comparisons. The chi-squared test was used to compare the subjective image noise in all groups, the visibility of anatomical structures in the 4 groups with different NI values, but independent of ASIR, and the other 4 groups with different ASIR values, but independent of NI. Chi-squared tests were performed on both the CT findings with different NI values and the detected numbers of lesions with different ASIR levels. A *P*-value of less than 0.05 indicates a statistically significant difference. Interobserver agreements were calculated using Cohen’s weighted Kappa statistics.

## Results

### Study Population and Radiation Dose

Imaging was performed on 200 patients. Each patient was scanned once, including the patients using NI = 40. Patient characteristics and radiation dose are shown in Table 1. No significant difference was found with respect to age, sex, height, weight, or BMI among the 4 groups. CTDI_vol_, DLP, and ED were significantly different in the four groups with different NI values. Compared to group 1, the mean values of CTDI_vol_ reduction were 39.93%, 72.96%, and 85.84%, and the percentages of ED reduction were 39.43%, 73.48%, and 86.74% in groups 2, 3, and 4, respectively.

**Table 1:** Patient characteristics and radiation dose.

Characteristics	Group 1	Group 2	Group 3	Group 4	*P*-value
	NI = 15	NI = 20	NI = 30	NI = 40	
Age (y)	56.14±14.64	53.53±11.79	50.86±11.76	50.12±10.60	0.07
M/F	37/13	34/16	31/19	28/22	0.27
Height (cm)	1.68±0.09	1.68±0.06	1.67±0.08	1.67±0.07	0.86
Weight (kg)	69.06±13.86	71.15±10.17	70.26±11.48	69.20±12.02	0.89
BMI (kg/cm^2^)	24.28±3.55	24.98±2.87	24.94±2.47	24.81±3.44	0.43
CTDI_vol_ (mGy)	5.51±2.21	3.31±1.18	1.49±0.53	0.78±0.49	<0.001
DLP (mGy˙cm)	193.59±81.10	117.28±40.64	51.29±19.93	25.78±15.12	<0.001
ED (mSv)	2.79±1.17	1.69±0.59	0.74±0.29	0.37±0.22	<0.001

Note: Data are presented as ratio of males/females or mean ± standard deviation. Each group had 50 patients.

### Assessment of Image Quality

**Figure 1:**
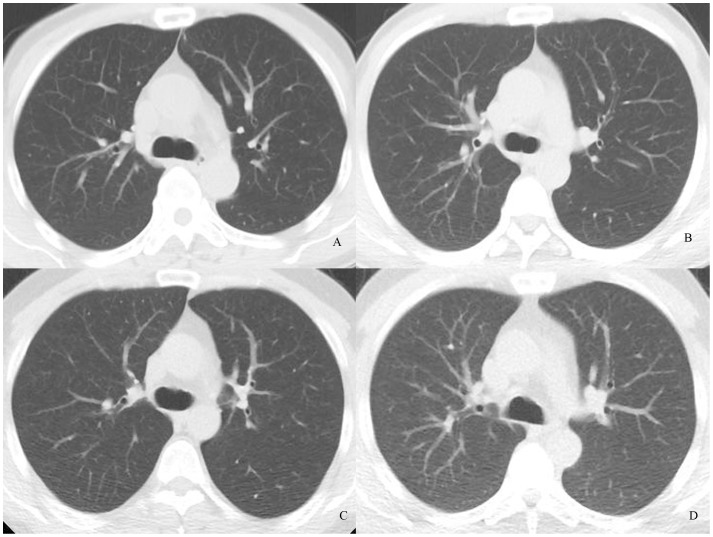
Transverse chest CT images of 4 patients who were almost the same height and weight. Images were reconstructed with 30% ASIR. Images are (A) NI = 15, objective image noise 10.95; (B) NI = 20, objective image noise 18.28, (C) NI = 30, objective image noise 28.5, and (D) NI = 40, objective image noise 41.52. The visibility of the small structure (arrow) in D was around average.

**Figure 2:**
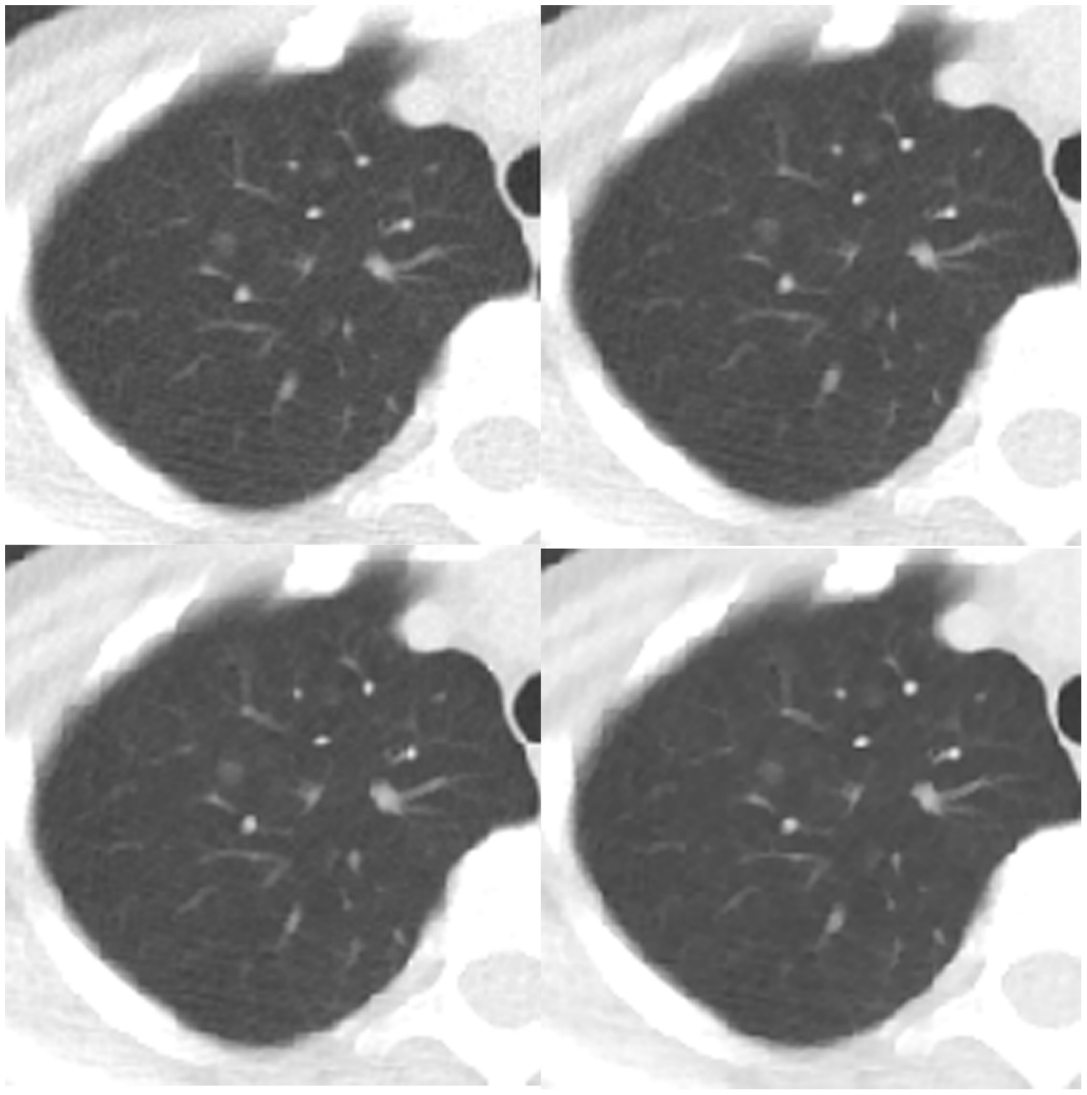
Transverse chest CT images of a patient who had a ground glass opacity nodule in the right upper lobe (arrow). Images are all NI = 30 and (A) 0% ASIR, objective image noise 32.37 HU; (B) 30% ASIR, objective image noise 25.90 HU, (C) 50% ASIR, objective image noise 22.15 HU, and (D) 80% ASIR, objective image noise 16.66 HU. The visibility of the small structure in D was higher than in A, and the margin of the lesion in D was clearer than in A.

Table 2 summarizes the number of pulmonary lesions identified at different NI settings, which were not significantly different from each other. Subjective visual lesion conspicuity was graded as 2 for 6 ground-glass opacity lesions at NI = 40 with 0% ASIR or 30% ASIR and 1 ground-glass opacity lesion at NI = 30 with 0% ASIR. Other lesions were graded as 1 out of 4 ASIR levels in each patient. Despite the grading differences mentioned above, the detected number of all lesions was not affected by different ASIR levels, as shown in Table 3. The subjective assessment scores of image quality of the pulmonary, mediastinal, and chest wall structures for the 4 groups are shown in Table 4. Increasing NI increased the subjective and objective image noise scores (*P*<0.001) (Fig. 1), as did decreased levels of ASIR (*P*<0.001) (Fig. 2). In contrast, SNR decreased with increasing NI (*P*<0.001), but increased with increasing ASIR (*P*<0.001) (Table 4).

**Table 2:** CT findings in groups with different NI values (n = 50 each).

CT finding	Group 1	Group 2	Group 3	Group 4	*P*-value
	NI = 15	NI = 20	NI = 30	NI = 40	
Normal	11	11	10	12	0.99
Emphysema	8	10	8	7	0.91
Solid nodule	19	19	21	18	0.96
Ground glass opacity nodule	5	3	5	5	0.88
Patchy consolidation	3	6	1	2	0.24
Patchy ground glass opacity	5	6	8	6	0.88
Scarring and calcification	24	21	23	23	0.96
Lesion of pleura	7	6	2	5	0.45
Other	2	2	2	3	1.00

Note: Data are shown as numbers of lesions; some patients had more than one kind of lesion.

**Table 3:** Number of detected lesions at different ASIR levels.

Lesion	0% ASIR	30% ASIR	50% ASIR	80% ASIR	*P*-value
Emphysema	33	33	33	33	1.00
Solid nodule	77	77	77	77	1.00
Ground glass opacity nodule	18	18	18	18	1.00
Patchy consolidation	12	12	12	12	1.00
Patchy ground glass opacity	25	25	25	25	1.00
Scarring and calcification	91	91	91	91	1.00
Lesion of pleura	20	20	20	20	1.00
Other	9	9	9	9	1.00

Note: Data are shown as numbers of lesions in 200 participants at different ASIR levels.

**Table 4:** Image quality assessment for chest CT images at 4 NIs with different ASIR levels**.**

NIvalue	Subjective imagenoise	Imageartifacts	Visibility of smallstructures	Visibility of mediastinaland chest wall structures	Objective imagenoise (HU)	SNR
0% ASIR						
15	1.72±0.45	1.13±0.34	1.72±0.45	1.63±0.49	16.12±1.35	2.57±0.43
20	2.67±0.47	1.16±0.37	2.28±0.45	2.48±0.50	22.61±2.68	1.85±0.40
30	3.53±0.50	1.17±0.38	2.80±0.40	3.17±0.55	33.83±3.86	1.20±0.21
40	4.22±0.42	1.24±0.43	3.63±0.54	4.17±0.49	47.01±5.57	0.96±0.18
30% ASIR						
15	1.52±0.50	1.17±0.38	1.59±0.49	1.58±0.50	13.69±1.02	3.02±0.50
20	2.11±0.37	1.16±0.37	2.30±0.63	2.29±0.67	18.50±2.23	2.27±0.50
30	3.22±0.52	1.19±0.39	2.57±0.54	3.08±0.60	27.51±2.90	1.47±0.25
40	3.92±0.71	1.21±0.41	3.33±0.70	4.04±0.49	37.83±4.40	1.19±0.22
50% ASIR						
15	1.26±0.44	1.17±0.38	1.60±0.49	1.61±0.49	11.99±1.03	3.44±0.53
20	1.78±0.42	1.20±0.40	2.18±0.64	2.10±0.54	16.00±1.92	2.61±0.55
30	2.88±0.33	1.17±0.38	2.48±0.50	2.95±0.46	23.42±2.47	1.73±0.32
40	3.49±0.50	1.29±0.46	3.17±0.47	3.81±0.39	32.27±3.71	1.40±0.25
80% ASIR						
15	1.07±0.26	1.66±0.48	1.50±0.50	1.27±0.45	9.77±1.18	4.26±0.76
20	1.20±0.40	1.81±0.39	1.93±0.54	1.89±0.57	12.51±1.74	3.32±0.84
30	2.07±0.33	1.84±0.37	2.42±0.62	2.58±0.64	17.90±2.21	2.27±0.41
40	3.04±0.42	1.90±0.30	3.04±0.58	3.66±0.55	24.37±2.97	1.84±0.33

Note: Data are shown as mean ± SD.

The subjective image noise scores were average in three subgroups (NI = 30 with 30% ASIR and 50% ASIR, NI = 40 with 80% ASIR), with objective image noise values ranging from 23.42 to 27.51 HU. Objective image noise scores in the NI = 30 and NI = 40 groups with different ASIR levels were similar, including: NI = 30 with 0% ASIR and NI = 40 with 50% ASIR (*P* = 0.42), NI = 30 with 50% ASIR and NI = 40 with 80% ASIR (*P* = 1.00), with no significant differences in SNR between these subgroups (Table 4). Subjective noise scores of the subgroups of NI = 30 with 0% ASIR and NI = 40 with 50% ASIR were higher than average. Mild pixilation artifacts were seen in subgroups with 80% ASIR regardless of the NI, but the artifacts did not interfere with diagnostic information. The 0% ASIR, 30% ASIR, and 50% ASIR groups had better image artifact scores than subgroups with 80% ASIR.

Both readers assessed anatomical structure visibility to be better than average or around average, except for the group of NI = 40, which had suboptimal scores for mediastinal and chest wall structures. The visibility for these anatomical structures improved with decreased NI (*P*<0.001). Visibility scores for these anatomical structures also improved with increased ASIR level, but did not vary significantly between 0% ASIR and 30% ASIR or 30% ASIR and 50% ASIR for both small and mediastinal and chest wall structures, or between 50% ASIR and 80% ASIR for only small structures. The interobserver agreements (kappa value) for image subjective noise, artifacts, visibility of small structures, visibility of mediastinal and chest wall structures of each subgroup were 0.616–0.802, 0.558–0.778, 0.406–0.606, and 0.472–0.700, respectively.

## Discussion

In this study, we analyzed the impact of NI and ASIR on image quality for low-dose chest CT screening. We could obtain quality images using an NI with ASIR algorithm and were able to reduce radiation doses to the sub-millisievert level.

Radiologists seek to reduce the radiation dose in CT screening, either by affecting the radiation dose directly via changing tube current, tube voltage, section thickness, scan length, NI, etc., or indirectly by using reconstruction algorithms. [26] Unfortunately, reducing the radiation dose will inevitably increase image noise and affect image quality. The conventional CT image reconstruction algorithm, FBP, reflects a trade-off between sharpness and image noise that limits the reduction of the radiation dose to maintain the diagnostic image quality. [27] ASIR is a newer image reconstruction algorithm that reduces image noise by applying iterations between the raw data and image space, generating images of higher quality and greater structural detail at lower radiation doses than FBP, [28] helping to improve image quality and reduce radiation dose.[17], [22], [23], [29]–[32] Iterative reconstruction algorithms could effectively reduce radiation doses for chest CT: one study showed a 27% radiation dose reduction using 30% ASIR [23], and another reported that higher ASIR levels (100%) could reduce radiation doses even further (76%). [33] ATCM is another method that we used to minimize radiation dose. Several studies indicated that CT dose indices could be reduced by 40–60% using ATCM without compromising image quality, [27], [34] and we could further reduce CT doses to sub-millisievert levels by using ATCM with ASIR.

To our knowledge, this is the first clinical study to evaluate the effects of different levels of ASIR on dose reduction in sub-millisievert low-dose chest CT screening by using ATCM for different NI settings. We were able to decrease the mean ED to 0.74 mSv at an NI of 30, and to produce minimal image artifacts, average subjective image noise, and nearly average anatomical structure visibility at NI = 30 with 50% ASIR. At these settings, the mean objective image noise was 23.42 HU, which is comparable to the result acquired at 40 mAs using 30% ASIR. [22] Similar subjective and objective image noise results were obtained by using NI = 40 with 80% ASIR or NI = 30 with 50% ASIR. The NI = 40 setting reduced the mean ED to 0.37 mSv, but produced suboptimal visibility of the mediastinal and chest wall structures, even though small structure visibility was sufficient, probably due to the high contrast of the small structures with air. The 80% ASIR produced more artifacts, regardless of NI, so we chose NI = 30 with 50% ASIR as the optimal settings for low-dose chest CT screening. The mean ED obtained using this setting was comparable with that from other studies. For example, one study reported a mean ED of 0.7 mSv for low-dose chest CT using iDose^4^ (an iterative reconstruction technique from Philips), [35] and another study reported a mean ED of 0.5 mSv for low-dose chest CT using SAFIRE (an iterative reconstruction technique from Siemens). [36] These are lower than the dose used in the NLST (1.5 mSv) and much lower than the dose used in standard chest CT (4–11 mSv). [14], [15], [29], [30].

The subjective and objective image noise and SNR significantly improved with increased ASIR; however, the visibility for the anatomical structures did not significantly improve between some adjacent ASIR levels, especially for the small structures, perhaps because image noise might not significantly impact the visibility of small structures that have high contrast with air. Similarly, the high contrast between air and lung lesions allowed for perfect lesion detection at all ASIR levels, as reported previously. [22] However, because of relatively the low contrast between ground glass opacity lesions and air, some lesions were observed with poorly visualized margins at lower doses with lower ASIR levels. The effects of lower radiation dose with different ASIR levels on such relatively low contrast lesions needs further evaluation with larger number of patients.

There are several limitations in our study. First, patient scans in the 4 groups were not acquired consecutively. However, there were no differences in patient characteristics (height, weight, BMI, age, and sex distribution) in the 4 groups. Second, the results only apply to this CT equipment because ASIR is vendor-specific. Third, ASIR is only the first generation of iterative reconstruction (IR); MBIR/Veo is an advanced algorithm that can reduce radiation dose more efficiently. [14], [15] However, MBIR/Veo is not currently available in our department. Fourth, we did not specify NI according to body size, which may further reduce radiation dose. [37].

### Conclusions

Combining ATCM with ASIR can effectively reduce radiation dose. A low-dose chest CT screening protocol of NI = 30 with 50% ASIR reduced the ED to 0.74 mSv while maintaining reasonable image quality.
